# NAD(H) Regulates the Permeability Transition Pore in Mitochondria through an External Site

**DOI:** 10.3390/ijms22168560

**Published:** 2021-08-09

**Authors:** Ekaterina Kharechkina, Anna Nikiforova, Alexey Kruglov

**Affiliations:** Institute of Theoretical and Experimental Biophysics, Russian Academy of Sciences, 142290 Pushchino, Russia; katya.kypri@gmail.com (E.K.); nikiforanna@yandex.ru (A.N.)

**Keywords:** permeability transition pore, NADH, NAD^+^, pore closure, calcium retention capacity, external regulatory site, cytosolic

## Abstract

The opening of the permeability transition pore (mPTP) in mitochondria initiates cell death in numerous diseases. The regulation of mPTP by NAD(H) in the mitochondrial matrix is well established; however, the role of extramitochondrial (cytosolic) NAD(H) is still unclear. We studied the effect of added NADH and NAD^+^ on: (1) the Ca^2+^-retention capacity (CRC) of isolated rat liver, heart, and brain mitochondria; (2) the Ca^2+^-dependent mitochondrial swelling in media whose particles can (KCl) or cannot (sucrose) be extruded from the matrix by mitochondrial carriers; (3) the Ca^2+^-dependent mitochondrial depolarization and the release of entrapped calcein from mitochondria of permeabilized hepatocytes; and (4) the Ca^2+^-dependent mitochondrial depolarization and subsequent repolarization. NADH and NAD^+^ increased the CRC of liver, heart, and brain mitochondria 1.5–2.5 times, insignificantly affecting the rate of Ca^2+^-uptake and the free Ca^2+^ concentration in the medium. NAD(H) suppressed the Ca^2+^-dependent mitochondrial swelling both in KCl- and sucrose-based media but did not induce the contraction and repolarization of swollen mitochondria. By contrast, EGTA caused mitochondrial repolarization in both media and the contraction in KCl-based medium only. NAD(H) delayed the Ca^2+^-dependent depolarization and the release of calcein from individual mitochondria in hepatocytes. These data unambiguously demonstrate the existence of an external NAD(H)-dependent site of mPTP regulation.

## 1. Introduction

Nicotinamide adenine dinucleotide (NAD(H)) plays a pivotal role in the regulation of a huge number of cellular processes. In addition to the participation in the transformation of energy, NAD^+^, and its derivatives regulate the activity and expression of numerous enzymes involved in cellular and mitochondrial metabolism, muscle contraction, DNA repair, antioxidant protection, biogenesis, cell differentiation and aging, autophagy, and inflammatory response [1,2,3,4,5,6]. Moreover, NAD(H) is one of the most potent endogenous regulators of the Ca^2+^-dependent mitochondrial permeability transition pore (mPTP) whose opening triggers cell death in various pathologies [7]. Earlier, it was shown that NADH at millimolar concentrations reversed the opening of mPTP and increased the capability of mitochondria to accumulate and retain Ca^2+^, acting on an unidentified regulatory site in the mitochondrial matrix [8,9,10,11]. NAD^+^ also induced the mPTP closure, but about 10 times less effective than NADH [8,9]. At the same time, the oxidation of endogenous NADH was associated with a decrease in the ability of mitochondria to accumulate and retain Ca^2+^, but not with the opening of mPTP per se [10,11]. The ability of added NAD(H) to close the opened mPTP was inhibited by added NADP(H), which was interpreted as a competition for the allosteric regulatory site [9]. In addition, the NAD^+^-dependent deacetylation of various proteins, in particular MnSOD, by SIRT3 [12,13] increased the resistance of mitochondria of aged rats to mPTP opening by Ca^2+^ [4]. Besides, the authors claimed that SIRT3 deacetylates the important mPTP promoter cyclophylin D; however, whether this affects the cyclophylin D activity or mPTP opening is currently unclear.

Until recently, there was little information about the regulation of mPTP opening by external (i.e., acting from the cytosolic side) NAD(H). On the one hand, it was established that the poly (ADP-ribose) polymerase-mediated degradation of cytosolic NAD^+^ is one of the main causes of cell death during ischemia/reperfusion [14,15,16,17]. Furthermore, increased cytosolic NADH/NAD^+^ ratios can be a signal for proliferation [18,19]. On the other hand, it was shown that external NADH can stimulate the mPTP opening in the presence of redox-cycling compounds such as menadione and lucigenin. The effect was mediated by NADH-dependent cytochrome *b*_5_ oxidoreductase 3 in the outer mitochondrial membrane (OMM), which reduced the redox cyclers to thiol-reactive radicals and thus promoted the mPTP opening [20,21,22].

Recently, we have found that NAD(H) added at physiological concentrations to the incubation medium (cytosolic side of mitochondrial membranes) potently suppresses the opening of mPTP in the mitochondria of differentiated cells and enhances the protective effect of adenine nucleotides and cyclosporin A (CsA) [23]. Since NAD(H) cannot penetrate through the intact inner mitochondrial membrane (IMM) [24,25], these data could indicate the existence of a NAD(H)-dependent site of mPTP regulation in the OMM or the intermembrane space (IMS) of mitochondria. However, it is well known that mPTP opening can be reversible [9,26,27,28,29], while exogenous NADH can penetrate through the opened mPTP and interact with matrix enzymes [30]. According to the earlier data of Haworth and Hunter, it was exogenous NAD(H) that caused the closure of the opened mPTP by acting on the allosteric regulatory site in the matrix [8,9]. Therefore, when registering a decrease in the optical density in mitochondrial suspension (which reflects the swelling of organelles), it is impossible to say, unambiguously, whether NAD(H) suppresses the opening or induces the closing of mPTP in each individual mitochondrion.

In the present work, we studied in detail the localization of the site of mPTP regulation by external NAD(H). We investigated the effects of external NAD(H) on the maximum CRC of mitochondria isolated from various organs, the swelling, and contraction of mitochondria in incubation media containing solutes transported and not transported by carriers of the IMM, and the Ca^2+^-dependent dissipation of the mitochondrial membrane potential (ΔΨm) and the release of 620-Da fluorescent dye from individual mitochondria of hepatocytes permeabilized with digitonin. The data obtained unambiguously indicate that the site of the regulation of mPTP by cytosolic NAD(H) is located either at the OMM, or the IMS, or the outer surface of the IMM.

## 2. Results

### 2.1. NAD(H) Increases the Ca^2+^-Retention Capacity of Liver, Heart, and Brain Mitochondria

**Figure 1:**
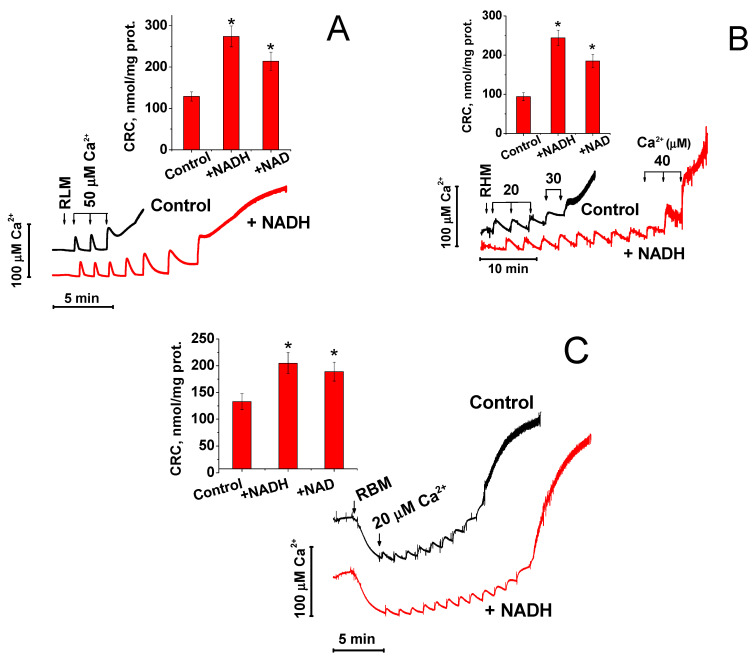
The effect of NADH and NAD^+^ on the CRC of the liver (**A**), heart (**B**), and brain mitochondria (**C**). Prior to the measurements, mitochondria (1 mg prot./mL) were added to the standard KCl-BM supplemented with 1 mM NADH or 1 mM NAD^+^. Arrows show the addition of Ca^2+^ by pulses of 20, 30, 40, and 50 µM. Representative curves of Ca^2+^ accumulation are shown for RLM (**A**), RHM (**B**), and RBM (**C**). Bars in the inserts are the average values of retained Ca^2+^ ± S.E.M (*n* = 6) for three or more independent experiments. * *p* < 0.01.

The release of Ca^2+^ from mitochondria can occur much earlier than the complete opening of mPTP and entry into the matrix of low-molecular-weight solutes (K^+^, Cl^−^, sucrose, mannitol, etc.), which cause the swelling of mitochondria [26,27,28,29]. The molecular weight of NAD(H) is significantly greater than the molecular weight of the hydrated forms of all the compounds mentioned above. Therefore, we first studied the effect of NADH and NAD^+^ on the maximum CRC of RLM, RHM, and RBM. As it follows from Figure 1, 1 mM NADH increased the CRC of RLM (A), RHM (B), and RBM (C) 2.1, 2.6, and 1.5 times, respectively, while 1 mM NAD^+^ was slightly less effective. The curves of Ca^2+^ uptake demonstrate that the protective effect of NADH was manifested before the massive release of accumulated Ca^2+^. Hence, NAD(H), presumably, protected mitochondria even before a significant increase in the permeability of the IMM.

### 2.2. Effect of NAD(H) on the Concentration of Ca^2+^ in the Medium and the Rate of Its Uptake by Mitochondria

**Figure 2:**
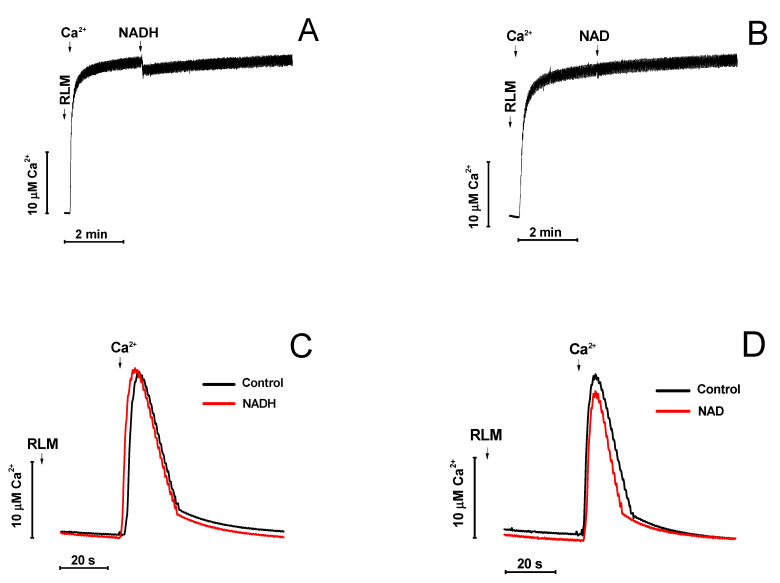
Effect of NADH and NAD^+^ on the concentration of free Ca^2+^ in the incubation medium (**A**,**B**) and the rate of Ca^2+^ uptake by mitochondria (**C**,**D**). (**A**,**B**). KCl-BM without mitochondria was placed in a chamber equipped with a Ca^2+^-selective electrode. Arrows indicate the addition of 20 µM Ca^2+^, 2 mM NADH (**A**), and 2 mM NAD^+^ (**B**). (**C**,**D**). RLM (1 mg prot./mL) were placed in the KCl-BM supplemented, where indicated, with 1 mM NADH (**C**) or 1 mM NAD^+^ (**D**). Two minutes later, 20 µM Ca^2+^ was added and its uptake was recorded. Representative curves of one experiment of at least three similar are shown.

Adenine nucleotides are capable of chelating Ca^2+^ in solution. We examined whether the protective effect of NADH and NAD^+^ is due to the binding of added Ca^2+^. Figure 2 shows that neither 2 mM NADH (A) nor 2 mM NAD^+^ (B) caused a significant decrease in the concentration of Ca^2+^ in the incubation medium. Voltage-dependent anion-selective channels (VDAC) in the OMM and the mitochondria calcium uniporter in the IMM are the main gates for Ca^2+^-uptake by mitochondria [31]. NAD(H) is known to be capable of binding to VDAC and modulate its permeability to small molecules [29]. Therefore, we studied the effect of NADH and NAD^+^ on the rate of Ca^2+^-uptake by mitochondria (Figure 2C,D). The figure demonstrates that neither NADH (C) nor NAD^+^ (D) inhibited the Ca^2+^ entry into mitochondria. Thus, it can be concluded that the protective effect of NADH and NAD^+^ on the induction of mPTP by Ca^2+^ cannot be explained by the chelation of Ca^2+^ or the inhibition of its transport into mitochondria by the nucleotides.

### 2.3. Divergent Effects of mPTP Closure on the Mitochondrial Shrinkage and ΔΨm Recovery in KCl- and Sucrose-Based Media

**Figure 3:**
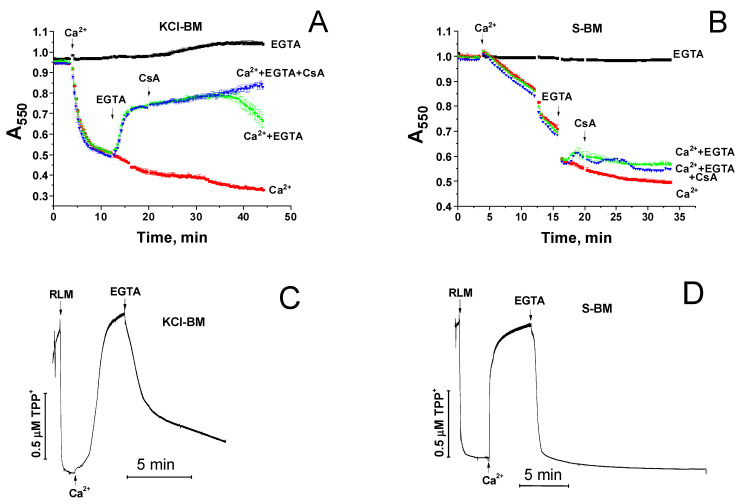
Effect of EGTA- and CsA-induced mPTP closure on the mitochondrial shrinkage (**A**,**B**) and ΔΨm recovery (**C**,**D**) in KCl-BM and S-BM. A and B. Just before measurements, RLM (0.75 mg prot./mL) were added to KCl-BM (**A**) or S-BM (**B**) supplemented, where indicated, with 1 mM EGTA. Arrows show the addition of 100 µM Ca^2+^, 1 mM EGTA, and 1 µM CsA. (**C**,**D**). Prior to the measurements, 1.5 µM TPP^+^ was added to KCl-BM (**C**) or S-BM (**D**). Arrows show the addition of RLM (1 mg prot./mL), 100 (**C**) and 400 µM Ca^2+^ (**D**), and 1 mM EGTA. Representative curves of one experiment of at least three similar are shown.

It was previously shown that the opening of mPTP in mitochondria can be reversible [9,32,33]. We studied the effect of mPTP closure by a Ca^2+^ chelator (EGTA) and an mPTP inhibitor (CsA) on the shrinkage and repolarization of mitochondria in incubation media whose particles can (KCl-BM) or cannot (S-BM) be transported by mitochondrial carriers across the IMM (Figure 3). In KCl-BM, Ca^2+^-dependent mitochondrial swelling (A) and a decrease in ΔΨm (C) was replaced by the shrinkage and restoration of ΔΨm when EGTA and CsA were added to the mitochondrial suspension. In S-BM, the addition of EGTA and CsA did not result in mitochondrial contraction (B), although the mPTP was closed, as is evident by the complete recovery of ΔΨm (D). Consequently, after the opening of mPTP and entry of sucrose molecules into the matrix, the swelling of mitochondria became irreversible even when the mPTP was closed. Taking this phenomenon into account, we developed an experimental model to find out the localization of the site of mPTP closure by added NAD(H).

### 2.4. Effect of NAD(H) on the Ca^2+^-Induced Mitochondrial Swelling in KCl- and Sucrose-Based Media

**Figure 4:**
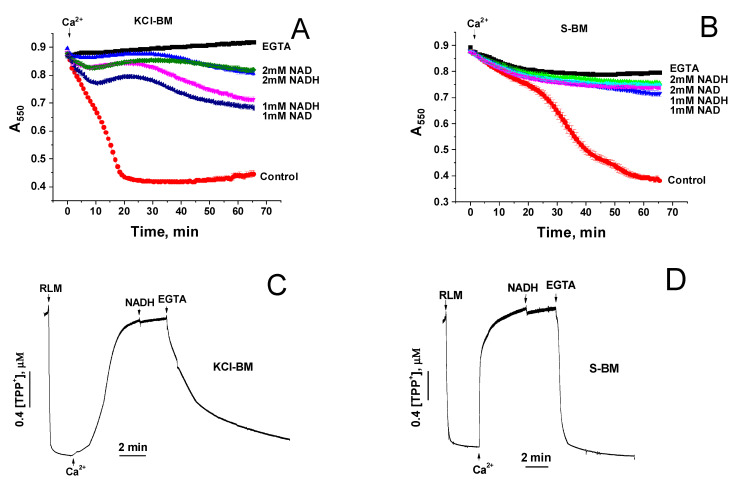
Effect of NADH and NAD^+^ on the Ca^2+^-induced mitochondrial swelling in KCl-BM (**A**) and S-BM (**B**). Prior to the measurements, RLM (0.75 mg prot./mL) were added to KCl-BM or S-BM supplemented, where indicated, with 1 mM EGTA and 1–2 mM NADH and NAD^+^. Arrows show the addition of 100 µM Ca^2+^. (**C**,**D**). Effect of NADH and EGTA on the recovery of ΔΨm in KCl-BM and S-BM. Just before measurements, 1.5 µM TPP^+^ was added to KCl-BM (**C**) or S-BM (**D**). Arrows show the addition of RLM (1 mg prot./mL), 100 (**C**) and 400 µM Ca^2+^ (**D**), 1 mM NADH, and 1 mM EGTA. Representative curves of one experiment of at least three similar are shown.

Based on the data presented in Figure 3, one can conclude that, if NAD(H) closes the mPTP by acting on the regulatory site in the matrix after passing through the pore, then in S-BM, in contrast to KCl-BM, the protective effect will disappear. Therefore, we examined the effect of added NADH and NAD^+^ on the rate of Ca^2+^-induced mitochondrial swelling in KCl-BM and S-BM (Figure 4). As it follows from Figure 3, in both KCl-BM (A) and S-BM (B), NADH and NAD^+^ effectively inhibited the mitochondrial swelling. Furthermore, the addition of NADH (C and D) and NAD^+^ (not shown) after the mPTP opening did not result in mitochondrial repolarization, whereas EGTA restored ΔΨm. These data directly indicate that NAD(H) added from the cytosolic side inhibits the opening rather than activating the closing of mPTP.

### 2.5. Effect of NAD(H) on the Ca^2+^-Induced Loss of ΔΨm and Calcein Release from Individual Mitochondria

**Figure 5:**
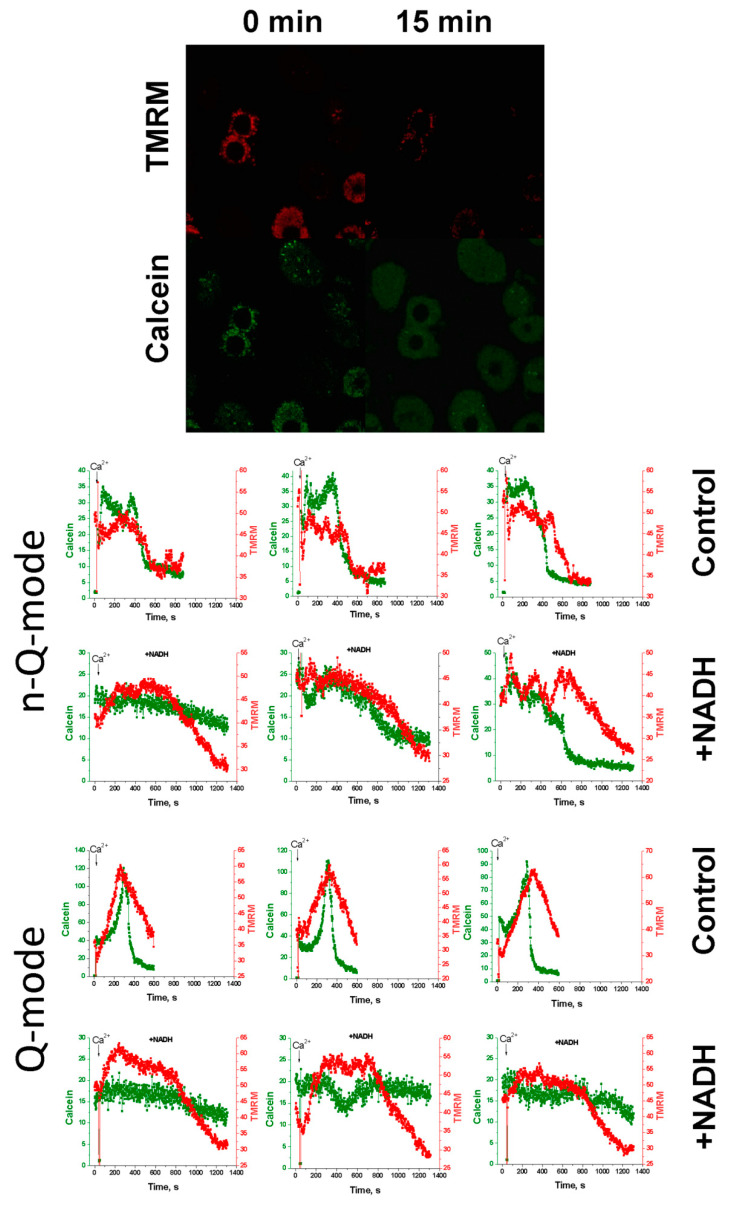
Effect of NADH on the Ca^2+^-induced loss of ΔΨm and calcein release from individual mitochondria. Isolated rat hepatocytes were stained with 5 µM calcein-AM and 100 (n-Q-mode) and 300 nM (Q-mode) TMRM. The cell incubation medium was replaced by mitochondria-supporting medium (125 mM KCl, 20 mM sucrose, 10 mM HEPES (pH 7.3), 5 mM succinate, 2.5 µg/mL rotenone, and 40 µM digitonin) supplemented, where indicated, with 2 mM NADH two minutes before the addition of 50 µM Ca^2+^. Representative traces of the dynamics of TMRM (red) and calcein fluorescence (green) in 12 selected mitochondria of 180 total from three separate experiments are shown.

There is a probability that external NAD(H) affecting the internal regulatory site through the opened mPTP causes its fast closure even before large changes in the mitochondrial volume. Since mPTP closure is not always accompanied by mitochondrial repolarization [29], one may suggest that NAD(H) induces mPTP closure without the restoration of ΔΨm, in contrast to the EGTA-induced one. To clarify this issue, we studied the effect of NADH and NAD^+^ on the dynamics of the Ca^2+^-dependent decrease in ΔΨm (TMRM) and the release of the fluorescent dye calcein from individual mitochondria of hepatocytes permeabilized with digitonin (Figure 5). Since the sizes of calcein and NAD(H) molecules are similar (620 and 663 Da, respectively), the beginning of the release of calcein from mitochondria corresponds to the moment when the membrane becomes permeable to NADH.

TMRM, at high concentrations, is known to quench the own fluorescence and the fluorescence of calcein (quenching mode, Q-mode) [34,35]. To prevent quenching, the concentration of TMRM in many studies was low (<40 nM) (non-quenching mode, n-Q-mode) [36,37]. However, during prolonged incubation, calcein may leak from mitochondria or undergo photobleaching, which interferes with the detection of the moment of mPTP opening. Therefore, a Q-mode sharp increase in the calcein fluorescence just before the opening of the pore [34,35] can be a convenient indicator of the initiation of the process (third line of panels). Figure 5 shows that, in the n-Q-mode (first and second lines of panels), NADH postponed the Ca^2+^-dependent high-amplitude drop in the TMRM and calcein fluorescence; in the Q-mode (third and fourth lines of panels), NADH delayed and decreased the amplitude of both the rise and decline in the TMRM and calcein fluorescence. Hence, NADH preserved ΔΨm and precluded the PTP opening in mitochondria of permeabilized hepatocytes. These data convincingly indicate that NAD(H) suppresses the PTP opening, acting through the external site in the OMM or the outer surface of the IMM.

### 2.6. Protective Effect of NAD(H) Is Due to the Suppression of Ca^2+^-Dependent Permeabilization of the Inner Mitochondrial Membrane

**Figure 6:**
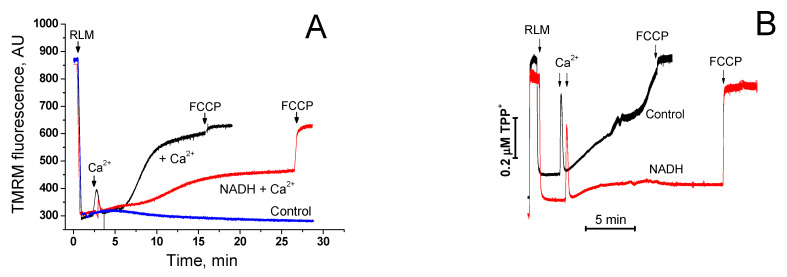
Effect of NADH on the Са^2+^-dependent dissipation of ΔΨm in mitochondrial suspension. Prior to the measurements, 150 nM TMRM (**A**) or 1 µM TPP^+^ (**B**) were added to KCl-BM. Arrows show the addition of RLM (0.3 (**A**) and 1 mg prot./mL (**B**), 50 (**A**) and 200 µM Ca^2+^ (**B**), and 500 nM FCCP. Each panel provides representative curves of one experiment of three similar.

One can propose two main mechanisms by which the external NAD(H) is capable of suppressing the Ca^2+^-dependent mitochondrial swelling: (1) a direct suppression of the mPTP opening through an external regulatory site and (2) suppression of the entrance of solutes into the intermembrane space and matrix by the VDAC blockage. In the second case, NAD(H) must not inhibit the Са^2+^-dependent dissipation of ΔΨm because the proton gradient is created across the IMM, whereas NAD(H) does not affect the rate of Ca^2+^ accumulation by mitochondria (see Figure 2). Figure 6 demonstrates that, in mitochondrial suspension, 1 mM NADH considerably delayed the Ca^2+^-dependent dissipation of ΔΨm measured by the membrane-penetrating lipophilic cations TMRM (A) and TPP^+^ (B). The addition of the uncoupler FCCP at the end of the measurements shows that NADH does not restrict the free passage of lipophilic dyes through the membranes. Hence, the data obtained indicate the existence of an external site of regulation of the mPTP state.

## 3. Discussion

It is well documented that pyridine nucleotides of the mitochondrial matrix are involved in the regulation of mPTP. NADH and NAD^+^ suppress the opening of mPTP by acting on the internal allosteric regulatory site [8,9] and increasing the CRC of mitochondria [10,11]. NADPH participates in the enzymatic reduction of glutathione, which, in turn, supports critical thiols of mPTP in a reduced state and thus suppresses the mPTP opening [10,11,38,39]. Mitochondrial transhydrogenase integrates the protective effects of NADH and NADPH, mediating the ΔΨm- and NADH-dependent reduction of NADP+ to NADPH [40].

Recently, we have shown for the first time that “external” or “cytosolic” NAD(H) can effectively suppress the opening of mPTP in mitochondria of terminally differentiated cells [23]. However, the experimental models used in the study left some possibility that external NAD(H) closed mPTP by acting on the internal regulatory site after the entry into the matrix through the open pore. In the present work, we provide unequivocal evidence for the existence of a NAD(H)-dependent “external” site of mPTP regulation located in the OMM, IMS, or at the outer surface of the IMM. The evidence is based on three effects of added NAD(H) on mitochondria: (1) on the increase in the CRC of isolated mitochondria (see Figure 1); (2) on mitochondrial swelling in sucrose- and KCl-containing media (see Figure 4); and (3) on the release of entrapped calcein from the mitochondria of permeabilized cells (see Figure 5).

The opening of mPTP is associated with a decrease in the ΔΨm, the release of accumulated Ca^2+^ and Mg^2+^ from mitochondria, and entry into the matrix of solutes with an average particle size of up to 1.5 kDa and even larger. The dissipation of ΔΨm, swelling in KCl-BM, Ca^2+^/Mg^2+^ release, and swelling in S-BM require pores with minimum radii of 0.14, 0.35, 0.42, and 0.52 nm, respectively, which correspond to the hydrodynamic radii of solvated particles [32,41,42]. However, the dissipation of ΔΨm and the release of Ca^2+^ and Mg^2+^ can be separated in time from the initiation of the sucrose and mannitol entry into the matrix by classical mPTP inhibitors (EGTA, CsA, ADP, Mg^2+^, and BSA) [29,43]. Moreover, the release of Ca^2+^ from mitochondria can occur upon the ΔΨm loss and oxidation of endogenous pyridine nucleotides without the mPTP opening [44,45,46]. Therefore, it is logical to assume that relatively large molecules such as NAD(H) (0.6 nm) will not be able to enter the mitochondrial matrix through the opened mPTP before a significant portion of the accumulated Ca^2+^ is released from mitochondria. It is reasonable that this should affect the kinetics of Ca^2+^ accumulation by mitochondria. Since we observed a NAD(H)-dependent increase in the number of loaded pulses of Ca^2+^ without any noticeable changes in the kinetics of the uptake (see Figure 1), this is a strong argument in favor of the existence of an external regulatory site.

Recording the Ca^2+^-dependent release of the ΔΨm-sensitive probe TMRM and the membrane-impermeable form of calcein from mitochondria by a confocal microscope makes it possible to directly determine the point in time when the internal regulatory site becomes accessible to the external NAD(H) (see Figure 5). In our experiments, we applied TMRM at two concentrations, which can (Q-mode) and cannot (n-Q-mode) quench the fluorescence of TMRM and calcein. It should be noted that usually lower TMRM concentrations are used in the studies of the PTP in cells [36,37]. However, the application of TMRM at higher concentrations has some advantages. Indeed, the quenching of TMRM and calcein fluorescence by high TMRM concentrations in the mitochondrial matrix terminates with the beginning of TMRM release, which facilitates the detection of the start of the mPTP opening [34,35]. According to our data, NADH considerably increased the time between the addition of Ca^2+^ and the release of calcein from mitochondria of hepatocytes and decreased the amplitude of fluctuations in ΔΨm. These data demonstrate that NADH entrance into the matrix is unnecessary for the mPTP inhibition.

The strongest evidence for the mPTP regulation through an external NAD(H)-binding site came from the data presented in Figure 3 and Figure 4. It is well known that the IMM contains specialized transport systems for the uptake and extrusion of K^+^ and Cl^−^ ions, but not for sucrose and NAD(H) [47,48,49,50]. Therefore, with the opening of mPTP (non-selective channel) in KCl-BM and S-BM, both ions and sucrose enter into the matrix, causing the mitochondrial swelling. The closure of mPTP in KCl-BM causes the IMM repolarization, pumping out of ions from the matrix, and the restoration of the mitochondrial volume. In S-BM, due to the absence of sucrose transport systems, the IMM repolarization occurs without the restoration of the mitochondrial volume (see Figure 3). Considering the size of sucrose and NAD(H) particles and their ratio in the incubation medium (500–125:1), one can assume that the accumulation of NAD(H) in the matrix sufficient for mPTP closure through the internal site must be accompanied by the accumulation of sucrose sufficient for high-amplitude swelling. Therefore, the effect of suppression of the mitochondrial swelling by the added NAD(H) should disappear in S-BM. The fact that NAD(H) demonstrates a potent protective effect in both KCl- and S-BM but fails to induce rapid repolarization of the IMM (see Figure 4) is direct evidence for the existence of an external NAD(H)-binding site for the mPTP regulation.

Presumably, the mechanism of mPTP suppression by external NAD(H) is distinct from the chelating of free Ca^2+^ by the nucleotide (see Figure 2). It was previously shown that the binding of NAD(H) to VDAC can restrict the movement of large (ATP/ADP) anions through the channel [50,51,52]. Other VDAC blockers inhibited the transport of small (superoxide) anions through the channel [53,54]. In addition, some data indicate that NADH and NAD^+^ can limit the permeability of the VDAC to Ca^2+^ and other small ions [32]. However, our experiments did not reveal any noticeable effect of NAD(H) on the rate of Ca^2+^ accumulation by mitochondria (see Figure 2), although the effect on the rate of mPTP induction (see Figure 4) and the CRC value (see Figure 1) was significant. Besides, the protective effect of NAD(H) does not appear to be associated with the restriction of the movement of incubation medium particles across the OMM, since the nucleotide suppressed both mitochondrial swelling and ΔΨm dissipation across the IMM (see Figure 6). Thus, the mechanism of NAD(H)-dependent inhibition of mPTP opening is most likely associated with pore stabilization in the closed state.

Despite the relatively low number of “external” NAD(H)-binding proteins that fit for the role of the mPTP regulator [23], some properties of the regulator extremely complicate its identification. Indeed, rather high effective concentrations of NADH and NAD^+^ for the inhibition of mPTP opening (IC50 ≥ 200 and 700 µM, respectively) indicate a low affinity of the regulator for the nucleotides. Besides, it is currently unknown whether the nicotinamide or adenine moiety of the NAD(H) molecule makes the major contribution to the mPTP suppression. In addition, integral membrane proteins require association with lipids to maintain their native structure and, hence, native structure for NAD(H) binding. All these peculiarities make the identification of the regulator by the immunoprecipitation method quite problematic. Earlier, we have supposed that the external mPTP regulator is one of the VDAC isoforms [30]. Indeed, VDAC can bind NADH and NAD^+^ with Kd of 87 and 680 µM, respectively [52,55]. Besides, VDAC is involved in the mPTP regulation [56] and interacts with other mPTP-regulating proteins: ANT, HKs I/II, and GAPDH [57,58,59,60,61]. However, regardless of the level of VDAC isoforms, the protective effect of NAD(H) in mitochondria of all cancer and undifferentiated cell lines we tested was absent or weak. This did not allow us to verify the hypothesis using the genetic knockout of candidate proteins. Thus, the molecular nature of the external NAD(H)-dependent mPTP regulator calls for further clarification.

To conclude, here we presented unequivocal evidence for the existence of an external mitochondrial NAD(H)-dependent site for the mPTP regulation. This finding is of considerable importance for the understanding of the mechanisms of mPTP control in vivo. Indeed, the existence of the site can explain the reasons for the additive inhibitory effects of cytosolic adenine and pyridine nucleotides on mPTP [23] and the resistance of mitochondria of excitable cells (cardiocytes, neurons) to long-term cyclic Ca^2+^ loads. In addition, the identification of the NAD(H)-binding regulator may allow the development of its high-affinity ligands for pharmacological stimulation or suppression of mPTP in various pathologies. Therefore, we believe that the results presented in this work will attract the attention of researchers and stimulate the development of this promising line of research.

## 4. Materials and Methods

### 4.1. Materials

Bovine serum albumin (BSA) (A2153), carbonyl cyanide p-(trifluoromethoxy)phenyl-hydrazone (FCCP) (C2920), CsA (30024), digitonin (D141), 4-(2-hydroxyethyl)piperazine-1-ethanesulfonic acid (HEPES) (H3375), NADH (N8129), NAD^+^ (N6522), rhodamine 123 (R8004), rotenone (R8875), sucrose (S7903), succinate (S3674), SF-6847/Tyrphostin A9 (T182), TPP^+^ (218790), Trizma Base (93352), were purchased from the Sigma-Aldrich Corporation (St. Louis, MO, USA). Ethylene glycol-bis(2-aminoethylether)-N,N,N′,N′-tetraacetic acid (EGTA) (A0878,0025) was from PanReac ApppliChem. Tetramethylrhodamine, methyl ester (TMRM), and Calcein-AM were from Thermofisher (Waltham, Massachusetts, USA). Cell-Tak (354240) (Corning, New York, NY, USA) was kindly provided by Dr. Polina Kotova from the ICB RAS. Other chemicals were of analytical grade and were purchased from local suppliers.

### 4.2. Isolation of Mitochondria from Rat Liver, Heart, and Brain

All manipulations with animals, before the isolation of the organs, were performed in accordance with the Helsinki Declaration of 1975 (revised in 1983), national requirements for the care and use of laboratory animals, and protocol 9/2020 of 17.02.2020 approved by the Commission on Biological Safety and Bioethics at the ITEB RAS. Adult male Wistar rats were decapitated after anesthesia with CO_2_. Rat liver mitochondria (RLM) were isolated by a standard differential centrifugation procedure [62]. The homogenization medium contained 220 mM mannitol, 70 mM sucrose, 10 mM HEPES (pH adjusted to 7.4 with Trizma Base), 1 mM EGTA, and 0.05% BSA. The mitochondrial pellet was washed three times with a medium devoid of EGTA and BSA. Final pellets were resuspended in this medium to yield ~70 mg protein/mL.

Rat heart mitochondria (RHM) were isolated in the same buffer in a similar way except the mitochondrial pellet was washed twice. The concentration of isolated RHM was approximately 20 mg protein/mL.

Rat brain mitochondria (RBM) were isolated as described in [63] with minor modifications. The homogenization medium contained 320 mM sucrose, 10 mM Tris (pH adjusted to 7.4 with Trizma Base), 0.5 mM EGTA and 0.5 mM EDTA. The brain was homogenized in a Potter homogenizer (800 rpm, 20 passages). The homogenate was centrifuged twice for 4 min at 2000× *g* and each time the precipitate was discarded. A “сrude” mitochondrial pellet containing synaptosomes, myelin, and non-synaptic RBM was obtained by sedimentation from supernatant at 12,500× *g* for 11 min. Then, RBM was purified by the sedimentation (17,500× *g* for 11 min) in a discontinuous Percoll gradient (3, 10, 15, and 24% Percoll in the isolation medium). The fraction of nonsynaptic mitochondria was collected and washed once in an isolation medium free of EGTA (11,500× *g*, 11 min). The concentration of isolated RBM was approximately 20 mg protein/mL.

Measurements were performed at 37 °C either in a KCl-based medium (KCl-BM) or in a sucrose-based medium (S-BM). KCl-BM contained 125 mM KCl, 20 mM sucrose, 10 mM HEPES (pH adjusted to 7.3 with Trizma Base), 2 mM KH_2_PO_4_, 2 mM MgCl_2_, 5 mM succinate, and 2.5 µM rotenone. S-BM was composed of 280 mM sucrose, 10 mM HEPES (pH 7.3), 2 mM KH_2_PO_4_, 2 mM MgCl_2_, 5 mM succinate, and 2.5 µM rotenone. Other experimental details are given in the figures and figure legends. Mitochondrial protein was assayed by the Biuret method using BSA as a standard [64].

### 4.3. Isolation of Rat Hepatocytes

Hepatocytes were isolated from 1 day-fasted rats (200–300 g) by collagenase digestion [65]. Livers were perfused with 0.3 mg/mL collagenase (Type IV) through the portal vein. Hepatocytes were separated from nonparenchymal cells by centrifugation at 50× *g* for 2 min at 4 °C. The viability of isolated hepatocytes was assessed by the trypan blue exclusion test and usually exceeded 80%. Isolated hepatocytes were stored in ice in Krebs-Ringer-HEPES buffer (130 mM NaCl, 3 mM KCl, 2 mM CaCl_2_, 1 mM KH_2_PO_4_, 1 mM MgSO_4_, and 20 mM HEPES (pH 7.35) supplemented with 0.2% BSA.

### 4.4. Determination of the Ca^2+^ Uptake Rate and the Ca^2+^-Retention Capacity of Mitochondria

Mitochondrial Ca^2+^ uptake and release were recorded in a temperature-controlled electrode chamber using a Ca^2+^-selective electrode (Niko Analyt, Moscow, Russia) connected to a computerized recording system Record 4 (ITEB RAS). The electrode was calibrated with known amounts of Ca^2+^ at the beginning of each experimental series. The CRC was defined as the amount of Ca^2+^ taken up by mitochondria in small pulses before the Ca^2+^ release. Other experimental details are given in the figure legends.

### 4.5. Recording of Mitochondrial Swelling

The opening of mPTP in isolated mitochondria was registered as the initiation of EGTA- and CsA-sensitive high-amplitude swelling. Mitochondrial swelling was determined by measuring a decrease in A_550_ in mitochondrial suspension using a plate reader (Infinite 200 Tecan, Groedig, Austria) and 96-well plates. Other details are given in the figures and figure legends.

### 4.6. Recording of the Mitochondrial Membrane Potential

ΔΨm across the IMM was measured using either an Infinite 200 plate reader or a TPP^+^-selective electrode (Niko Analyt, Moscow, Russia) connected to the recording system Record 4. For fluorescent and potentiometric measurements, incubation media contained either 330 nM rhodamine 123 (Ex 480 nm, Em 525 nm) or 1.5 µM TPP^+^.

### 4.7. Registration of mPTP Opening by Confocal Microscopy

Hepatocytes were stained with 5 µM calcein-AM and 100 (non-quenching mode) or 300 nM (quenching mode) ΔΨm-sensitive dye TMRM at 37 °C for 15 min. Then, hepatocytes were set on a glue (Cell-Tak), and the Krebs-Ringer-Hepes buffer was gently washed out and replaced by KCl-BM supplemented with digitonin (40 µM/(mL∙10^6^ cells)), and, where specified, NADH and NAD^+^ at indicated concentrations. The dissipation of ΔΨm and the opening of mPTP in selected mitochondria were recorded at 540/517–600 and 488/520–570 nm Ex/Em, respectively, as described by Hüser et al. [34,35].

### 4.8. Statistical Analysis

All original curves are representative of one experiment of at least three similar. The values on all swelling/shrinkage and ΔΨm curves measured by rhodamine 123 are the means ± standard error of the mean (S.E.M.) for three wells (*n* = 3). Bars are the means ± S.E.M. from three or more experiments. Statistical significance (*p*) was determined using the Student’s *t*-test.

## Data Availability

Data is contained within the article.
